# Understanding a Core Pilin of the Type IVa Pili of *Acidithiobacillus thiooxidans*, PilV

**DOI:** 10.4014/jmb.2310.10033

**Published:** 2024-01-17

**Authors:** Araceli Hernández-Sánchez, Edgar D. Páez-Pérez, Elvia Alfaro-Saldaña, Vanesa Olivares-Illana, J. Viridiana García-Meza

**Affiliations:** 1 Geomicrobiología, Metalurgia, Universidad Autónoma de San Luis Potosí, Sierra Leona 550, San Luis Potosí, 78210, SLP, México; 2 Laboratorio de Interacciones Biomoleculares y Cáncer. Instituto de Física, Universidad Autónoma de San Luis Potosí, Av. Parque Chapultepec 1570, Privadas del Pedregal, San Luis Potosí, 78210, SLP, México

**Keywords:** PilV, protein expression, protein purification, protein structure, protein disorder, *A. thiooxidans*

## Abstract

Pilins are protein subunits of pili. The pilins of type IV pili (T4P) in pathogenic bacteria are well characterized, but anything is known about the T4P proteins in acidophilic chemolithoautotrophic microorganisms such as the genus *Acidithiobacillus*. The interest in T4P of *A. thiooxidans* is because of their possible role in cell recruitment and bacterial aggregation on the surface of minerals during biooxidation of sulfide minerals. In this study we present a successful *ad hoc* methodology for the heterologous expression and purification of extracellular proteins such as the minor pilin PilV of the T4P of *A. thiooxidans*, a pilin exposed to extreme conditions of acidity and high oxidation-reduction potentials, and that interact with metal sulfides in an environment rich in dissolved minerals. Once obtained, the model structure of *A. thiooxidans* PilV revealed the core basic architecture of T4P pilins. Because of the acidophilic condition, we carried out *in silico* characterization of the protonation status of acidic and basic residues of PilV in order to calculate the ionization state at specific pH values and evaluated their pH stability. Further biophysical characterization was done using UV-visible and fluorescence spectroscopy and the results showed that PilV remains soluble and stable even after exposure to significant changes of pH. PilV has a unique amino acid composition that exhibits acid stability, with significant biotechnology implications such as biooxidation of sulfide minerals. The biophysics profiles of PilV open new paradigms about resilient proteins and stimulate the study of other pilins from extremophiles.

## Introduction

Pili are polymers of pilins, dynamic semiflexible filaments anchored to and extending from the cellular membrane of most Bacteria and Archaea. They are about 4-9 nm in diameter and 4-5 μm long but can be extended by up to several more micrometers by adding additional units (polymerize) and retracted (depolymerize) by removing units. These units are generically named pilins [1-4]. Extension or retraction can occur at a rate of >1000 subunits/s [5]. The complex pilin assembly process employs a proteinaceous machinery that uses the energy required for assembly and disassembly provided by two cytoplasmic ATPases associated with the transmembrane complex of the pilus [1, 6]. Thus, the basic pilins are: a major pilin, adhesion pilin, minor pilins, and assembly and retraction ATPases [5, 7]. Pili play a number of different roles in cells, such as motility, chemotactic migration, surface sensing, surficial twitching motility, detection of neighboring cells, microcolony and biofilm formation, secretion of colonization factors, resistance to mechanical stresses >100 pN [8,9], cell aggregation, stabilization of extracellular polymeric substances (EPS), adhesion to surfaces or to host cells [10], and enabling extracellular electron transport [11-14] and DNA uptake and lateral gene transfer [9], among other functions.

Various types of pili have been described according to their mechanisms of assembly, pilin structure, and morphology [1]. The type IV pili (T4P) are the most common filaments or fimbriae [15] and have been grouped based on the aminoacidic homology of the pilins, which are relatively conserved in Gram-negative bacteria [16, 17], including chemolithoautotrophic acidophiles such as *Acidithiobacillus ferrooxidans* and *A. thiooxidans* [11, 18-20]. All T4P share domain structures and preserved sequences such as the conserved N-terminal motif in major and minor pilins, which is a type III signal peptide [1, 6, 21]. This motif is cleaved off by one peptidase before the mature protein is incorporated into the pilus structure [22] and it has become the landmark for pilus structure proteins [23, 24].

The structure of T4P and their pilins in pathogenic bacteria are well characterized; however, little is known about the T4P proteins or pilins in acidophilic microorganisms such as the genus *Acidithiobacillus*. The interest in *A. ferrooxidans* and *A. thiooxidans* is because of their role in bioleaching during metal removal in landfills and mining. To access the free electrons from sulfide minerals such as pyrite (FeS_2_), chalcopyrite (CuFeS_2_) or sphalerite (ZnS), both acidithiobacilli require contact with the mineral surface, which allows individuals to begin to form biofilms. Previous research has shown that the pili of *A. ferrooxidans* are involved with recruitment of cells and bacterial aggregation to the surface of pyrite, and a significant up-expression of pilins involved in cell adhesion and pili biogenesis has been detected [12, 18]. Also, for *A. ferrooxidans*, T4P enable electron transference in addition to their adhesion properties [11, 13]. Like *A. ferrooxidans*, surface colonization and adhesion by *A. thiooxidans* should be mediated by pili and enhanced by EPS. Thus, a better understanding of the role of pilins during the colonization of mineral surfaces can improve the mineral bioleaching processes employed to recover Cu, Ni, Zn, Co and/or Au, or to improve sulfur removal from solid sources, and even energy generation in microbial fuel cells [25, 26]. It is important to note that all of these bioleaching processes occur at low pH values (0.5-2.5) and under high oxidant conditions.

In some proteobacteria such as *Pseudomonas* spp. and *Geobacter* spp., PilV is a minor pilin or outer membrane protein necessary for pilus biogenesis and stabilization [27, 28]. PilV acts after pilus assembly along with other minor pilins (*e.g.*, PilW, PilX, PilY1), forming a core assembly initiation subcomplex that primes pilus assembly by lowering the energy barrier necessary for major pilins to emerge from the membrane, in which PilV interacts with FimU, which then interacts with major PilA [28, 29]. PilV includes the consensus motif or signal peptide sequence GXXXXE, as well as a highly conserved N-terminal domain in bacterial T4P pilins, whose functions are inner membrane insertion, signal processing, and pilin polymerization to form a central helical core of the pilus filament [27, 30]. PilV has been described in *A. ferrooxidans* as a “prepilin-type N-terminal cleavage/methylation domain-containing protein”, domain that belongs to the PilV superfamily. An extracellular structure, the T4P pilus assembly protein involved in cell motility, e.g., lower expression PilV mRNA in *A. ferrooxidans*, affects motility and attachment to the substrate [31]. What is interesting is that the PilV of *Acidithiobacillus* species has been little described and analyzed. PilV of *A. thiooxidans* shares up to 93% homology with PilV of *A. ferrooxidans* in its mature form [20]. Recently we found that PilV is in a cluster *fimT-pilW-pilV-pilX-pilY1* similar to the minor-pilins cluster of *P. aeruginosa* [32]. It is clear that pathogenic and acidophilic bacteria have type IV pili (T4P); therefore, their pili are in contact with different external environments. It is possible that among all types of pili in different microorganisms, the pilins share shape and size properties. However, at the same time, they should present differences at the surface level that enable them to adapt to thousands of different external conditions.

In the realm of acidophilic prokaryotic organisms, maintaining homeostasis assumes paramount importance for their survival. Numerous studies have concentrated on elucidating intracellular and extracellular mechanisms of resistance to low pH, with references to recent work by Feng *et al*. [33], Boase *et al*. [34], and González-Rosales *et al*. [35]. However, scant attention has been paid to the adaptations of extracellular protein structures, such as T4P, which remain vulnerable to low pH due to their exposure outside the cell. Recent studies have hinted at potential mechanisms for acid protection in T4P. In acidophilic thermophilic Archaea, the prevalence of amino acids like serine and threonine is thought to provide acid protection, potentially via O-glycosylation sites, as proposed by Wang *et al*. [36]. On the other hand, bacterial T4P also exhibit features that suggest acid stability, characterized by a composition rich in polar amino acids and a low proportion of charged amino acids, as discussed in a recent study by Páez-Pérez *et al*. [32]. Similar trends in amino acid composition have been observed in pili-like structures in *A. ferrooxidans*, most likely reflecting a responsive variation linked to the protein stabilization at low pH [37]. These findings shed light on the fascinating adaptations of extracellular protein structures in acidophilic microorganisms, offering insights into their ability to thrive in challenging acidic environments.

Currently there are no reports regarding cloning and heterologous expression of major or minor pilins from species of *Acidithiobacillus* despite their being acidophiles, nor are any reports of *in silico* or experimental analysis pilins from acidophiles, *e.g.*, the acid stability of their pilins. Here we introduce such analyses with the premise that the pilin PilV is due to a particular amino acid composition indicating a reduction in charged amino acids. This was computationally evaluated using molecular dynamics and stability predictions, which showed acid stability, likely due to low surface charge. Also, in this work, PilV of *A. thiooxidans* was expressed and purified for the first time. Through molecular cloning techniques, recombinant proteins were obtained by *in vitro* expression systems. Cloning was done using the TOPO-TA system (vector pGEM-T Easy), and its insertion into *E. coli* JM109; the *pilV* obtained from the pGEM-*pilV* construct was ligated into the expression vector pET-32b(+), then expressed in *E. coli* BL21-CodonPlus (DE3) and purified with nickel affinity and anion exchange chromatography. The stability of the protein at different pH was then evaluated by monitoring its solubility using UV-Vis and fluorescence spectroscopy.

## Material and Methods

### Culture of *Acidithiobacillus thiooxidans* and Synthesis of cDNA Pilin

Cells of type strain *A. thiooxidans* ATCC 19377 were aerobically cultured at 29 ± 1°C in ATCC-125 medium (with g/l: 0.4 (NH_4_)_2_SO_4_, 0.5 MgSO_4_·7H_2_O, 0.25 CaCl_2_, 3 KH_2_PO_4_, 0.005 FeSO_4_·7H_2_O; 10 of sterilized S^0^; pH 2.0 adjusted with concentrated H_2_SO_4_). The cultures were incubated for 28 days to obtain *ca*. 1 × 10^8^ cells/ml. At this cellular density, the cells were concentrated and washed at 800 ×*g* for 1 min to remove the S^0^, and at 21,100 ×*g* for 1 min using saline phosphate buffer PBS; in g/l: 8 NaCl, 1.44 Na_2_HPO_4_, 0.24 KH_2_PO_4_ (J.T. Baker, USA), and 0.2 KCl (Sigma, USA; pH 7.4). The bacterial pellet was preserved until its use. mRNA was extracted by the TRIzol (Invitrogen, USA) method following the protocol provided by the manufacturer and the cDNA was obtained using M-MLV retrotranscriptase (RT, Invitrogen).

### Design of Primers and PCR

Primers were designed based on the sequence of a non-redundant PilV protein (AWP39907.1; GenBank, ncbi.nlm.nih.gov/genbank) of *A. thiooxidans* ATCC 19377 (NZ_SZUV01000001.1:273124-274257; GenBank), using Vector NTI Express (Thermo Fisher Scientific). The primers were then synthesized by T4 Oligo (México) as follows: *pilV* forward: 5'-CTCACTCTCATTGACTATGATCG-3'; *pilV* reverse, 5'-TCAGTATCCCACGATGGTTTG- 3'. We obtained the PCR-amplified region of 444 bp of *pilV*.

The *pilV* mRNA was amplified using GoTaq DNA Polymerase (Promega, USA) according to the protocol provided by the manufacturer. The PCR cycling conditions were: Initial denaturation (95°C, 1 min), 35 cycles consisting of denaturation (95°C, 1 min), primer annealing (55°C, 1 min), and extension (72°C, 1:20 min); followed by a final extension step (72°C, 5 min). PCR products were analyzed by electrophoresis in 1% (w/v) agarose gels (Sigma) and purified by gel extraction with the Wizard SV Gel kit and PCR Clean-Up system (Promega).

### Cloning of *pilV*

The purified *pilV* product of PCR was ligated into the pGEM-T Easy cloning vector (Promega) at the following proportions: 5 μl of buffer ligation 2X, 1 μl of pGEM-T Easy vector, 1-3 μl of PCR product, 1 μl of T4 DNA ligase (Promega), and DNAse-free water, incubated 1 h at room temperature. After ligation, the *E. coli* JM109 competent cells (Promega) were transformed according to the protocol provided by the manufacturer. *E. coli* strains were inoculated at 37°C into Luria-Bertani (LB) medium supplemented with ampicillin (100 μg/ml; Sigma-Aldrich), IPTG (Isopropylthio-β-D-galactoside) (0.5 mM; Invitrogen, USA) and X-Gal (5-bromo-4-chloro-3-indolyl-β-d-galactopyranoside) (50 μg/ml; Roche, Germany).

Minipreps of transformed cells were carried out using the QIAprep Spin Miniprep Kit (Qiagen, Germany), followed by sequencing analyses using the universal M13 primers of the pGEM-T Easy vector, to confirm the correct direction of *pilV* insertion. The sequences of the inserts were analyzed at LANBAMA (National Biotechnology Laboratory, IPICYT, México). The recombinant plasmid was named pGEM-*pilV*.

The pGEM-*pilV* and the pET-32b(+) vector (Novagen, USA) were digested with EcoRI (Invitrogen) at 37°C for 1 h and the enzyme was then inactivated by incubating at 65°C for 15 min; the resulting fragments were separated by electrophoresis in 1% (w/v) agarose gels and purified by gel extraction (Wizard SV Gel and PCR clean-up system; Promega).

### Subcloning the *pilV* Fragment into pET-32b(+) Expression Vector

We chose pET-32b(+) as the expression vector because it provides a higher level of expression than other systems and because the proteins are fused with a thioredoxin (Trx) that favors protein solubility, avoiding the formation of inclusion bodies [38]. The *pilV* fragment was subcloned into the pET-32b(+) vector (Novagen, Germany) using T4 DNA ligase (Promega) with an insert:vector ratio of 3:1; the reaction was carried out at room temperature for 1 h. The resulting pET32-*pilV* construct was sequenced for verification, and then transformed into *E. coli* Top10 (Invitrogen) according to the manufacturer’s protocol. The cells were incubated on ice for 30 min followed by a thermal shock in a water bath at 42°C for 30 s. The cells were then incubated at 37°C for 24 h in LB medium. The transformed cells were then collected and cultured in solid LB medium supplemented with ampicillin (50 μg/ml; Sigma-Aldrich), at 37°C for 16 h. The pET32-*pilV* construct was extracted from the transformed cells by means of minipreps and digested with EcoRI as previously described. The presence, integrity, and orientation of the inserts were verified by PCR using the universal T7 primers and sequencing (LANBAMA, Ipicyt, Mexico). The correctly oriented clones were stored at -20°C until use.

### PilV Expression and Purification

The fusion protein was expressed in *E. coli* BL21-CodonPlus (DE3) cells (Agilent Technologies, Inc. 2015) as follows: first, fresh bacterial colony harboring the recombinant vector was inoculated into 500 ml of LB medium with 100 μg/ml ampicillin, 75 μg/ml streptomycin and 34 μg/mL chloramphenicol and incubated at 37°C until it reached an absorbance of 0.8 at 600 nm (OD_600_). Then the inductor (IPTG) was added to a final concentration of 1.0 mM; the culture was orbitally incubated at 16°C overnight, and lastly, the cells were harvested by centrifugation at 4,200 ×*g* for 10 min [39] and the pellet was stored at -20°C until use.

Bacteria were resuspended in 30 ml of buffer A (50 mM HEPES pH 7.0, 100 mM NaCl and 0.1 mg/ml lysozyme) and disrupted by sonication (amplitude 50%; pulse on 15 s; pulse off 45 s). Soluble and insoluble fractions were separated by centrifugation at 15,000 ×*g* for 30 min at 4°C and aliquots from the supernatant and pellet were used to determine recombinant protein expression by sodium dodecyl sulfate polyacrylamide gel electrophoresis (SDS-PAGE) using Coomassie brilliant blue (BioRad, Switzerland) for staining. The recombinant fusion protein was found in the insoluble fraction.

The insoluble fraction was resuspended in buffer B (20 mM Tris-HCl pH 8.0, 2% (v/v) Triton X-100 and 1 M urea) and stirred for 30 min, then centrifuged at 15,000 ×*g* for 30 min at 4°C, recovering the fusion protein from the supernatant.

The His6 tagged fusion protein was purified by a one-step procedure. The supernatant was loaded onto a column with His60 Ni-Superflow resin (Takara, USA) equilibrated with buffer B. The column was washed with three column volumes (CVs) of buffer C (20 mM Tris-HCl pH 8.0, 2% Triton X-100 and 100 mM NaCl). Subsequently the column was washed extensively with buffer C supplemented with 20 mM imidazole. Elution of the recombinant protein was carried out in one step with three CVs of buffer C supplemented with 300 mM imidazole.

To verify protein expression and purification, SDS-PAGE was carried out with 12% acrylamide gels using established procedures [40].

Gels for western blot (WB) were transferred to 0.45 μm nitro-cellulose membrane (Bio-Rad, Switzerland) at 300 mA for 1 h. The membranes were blocked for 1 h in PBS 1× with 5% (w/v) non-fat milk and 0.01% Tween 20, and then incubated at 4°C overnight with mouse 6x-His Tag (1:1000; Invitrogen). After this, the membrane was washed (three times, each time for 10 min in agitation) with TBS 1× with 0.01% Tween 20, incubated with mouse Anti-His-HRP secondary antibody (1:5000; Invitrogen) for 1 h, at room temperature and washed as previously described.

In order to obtain free PilV in solution, digestion was carried out with thrombin protease, for which we used recombinant thrombin from Sigma-Aldrich according to the manufacturer’s instructions.

After digestion, the sample was desalted in 25 mM Tris pH 8.0, 150 mM NaCl, 2% Triton X-100, and applied to an anion exchange column (Pierce Strong Anion Exchange Spin Columns), which was extensively washed with the same buffer. Subsequently, the protein was eluted with a buffer containing 25 mM Tris-HCl pH 8.0, 300 mM NaCl, and 2% CHAPS. The protein was quantified by measuring absorbance at 280 nm, and it was stored at 4°C until further use.

### Structure Prediction and Structure Alignment Analysis

For prediction of the PilV structures of *A. thiooxidans* ATCC 19377 and other bacteria (*Thermus thermophilus* HB27, *Pseudomonas aeruginosa* PAO1 and *Geobacter sulfurreducens* PCA), the AlphaFold2 program ColabFold V1.5.2 was used (https://alphafold.ebi.ac.uk/) following the instructions recommended by Jumper *et al*. [41]. The complete amino acid sequence of pilins, including the propeptide, were submitted and analyzed with default parameters to run in each prediction. Using PilV structures obtained in PDB format generated by AlphaFold2, its structures as well as structure alignment analyses and the percent of similarity (two by two) were visualized and computed using UCSF Chimera 1.16 (www.cgl.ucsf.edu/chimera/) [42].

### Disorder, Secondary Structure, and Transmembrane Topology Prediction

Structural disorder was predicted using the PONDR server (http://www.pondr.com/) [43], employing the VL-XT algorithm, PSIPRED (http://bioinf.cs.ucl.ac.uk/psipred/) [44], and DeepTMHMM (https://dtu.biolib.com/DeepTMHMM/) [45].

### Protonation and Deprotonation of PilV at Different pH

The protonation status of acidic and basic residues in the PilV protein was analyzed using the PDB2PQR web server (https://server.poissonboltzmann.org/pdb2pqr). The PDB2PQR uses the PROPKA algorithm together with the PARSE force field to calculate the ionization state of the protein at specific pH values. So, the submitted PilV structure was modified by incorporating protons, which were added according to the ionization states of the titratable groups at pH 2, 3, 4, 5, 6, 7, and 8. The Protein-sol program [46] (https://protein-sol.manchester.ac.uk/) was used to evaluate the pH stability of PilV. This program computes the stability of the folded conformation and the charge characteristics of the proteins as pH varies. It employs the Debye-Hückel formula to determine the pKa, thus accounting for the pH-dependent impact on the stability of the folded state.

### Molecular Dynamics Simulation

Molecular dynamics (MD) simulations of the different protonation states of PilV that had been generated were carried out using the WebGRO program, based on GROMACS (https://simlab.uams.edu/index.php). To assess the stability of PilV, four main parameters were evaluated: RMSD (root mean square deviation), RMSF (root mean square fluctuation), Rg (radius of gyration) and total number of hydrogen bonds. The force field used was GROMOS96 43a1. Simple Point Charge (SPC) was selected as the solvent model (triclinic water box). This system was neutralized by adding sodium or chlorine ions depending on the total charges. Protein energy minimizations were performed using the steepest descent method in the presence of solvent, with a maximum of 5000 steps. MD simulations were carried out in the presence of 0.15 M NaCl using a constant temperature (300 K) and pressure (1.0 bar). Approximately 1000 frames were generated each simulation. The simulation time was set to 50 ns.

### pH-Dependent PilV Aggregation

To measure precipitation propensity, PilV was suspended in 10 mM sodium phosphate buffer at the specified pH. After centrifugation for 20 min at 20,000 ×*g*, the absorbance at 280 nm was measured using a Nanodrop spectrophotometer. Samples were kept at room temperature between measurements. Extinction coefficients obtained from ExPASy ProtParam (https://web.expasy.org/protparam/) [47] were employed to convert absorbance into protein concentration.

### Exposure of PilV to Different pH and Denaturing Conditions

The PilV protein was diluted to 5 μM using different buffers at various pH values (2.0, 3.6, 5.0, and 8.0) and equilibrated at room temperature. The following buffers (100 mM each) were used: glycine-HCl (pH 2.0), acetate (pH 3.6 and 5.0) and sodium phosphate buffer (pH 8.0).

A 5 μM PilV sample was diluted in 8 M urea and incubated at room temperature for 60 min. For heat denaturation, another 5 μM PilV sample was diluted in water and incubated at 90°C for 15 min.

### Fluorescence Spectroscopy

The fluorescence emission spectra of PilV (5 μM) at various pH levels and denaturing conditions were measured using a Cary Eclipse Fluorescence Spectrophotometer from Agilent Technologies in a quartz cuvette with a 1 cm path length. The samples were excited at 280 nm, and the fluorescence spectra were recorded within the range of 300–400 nm. Both the excitation and emission slit widths were fixed at 5 nm. The samples were incubated in the dark at room temperature for 5 min before recording the fluorescence emission spectra. All values are the mean of three independent measurements.

## Results

### Inserts Obtained after Cloning Protocol

**Fig. 1:**
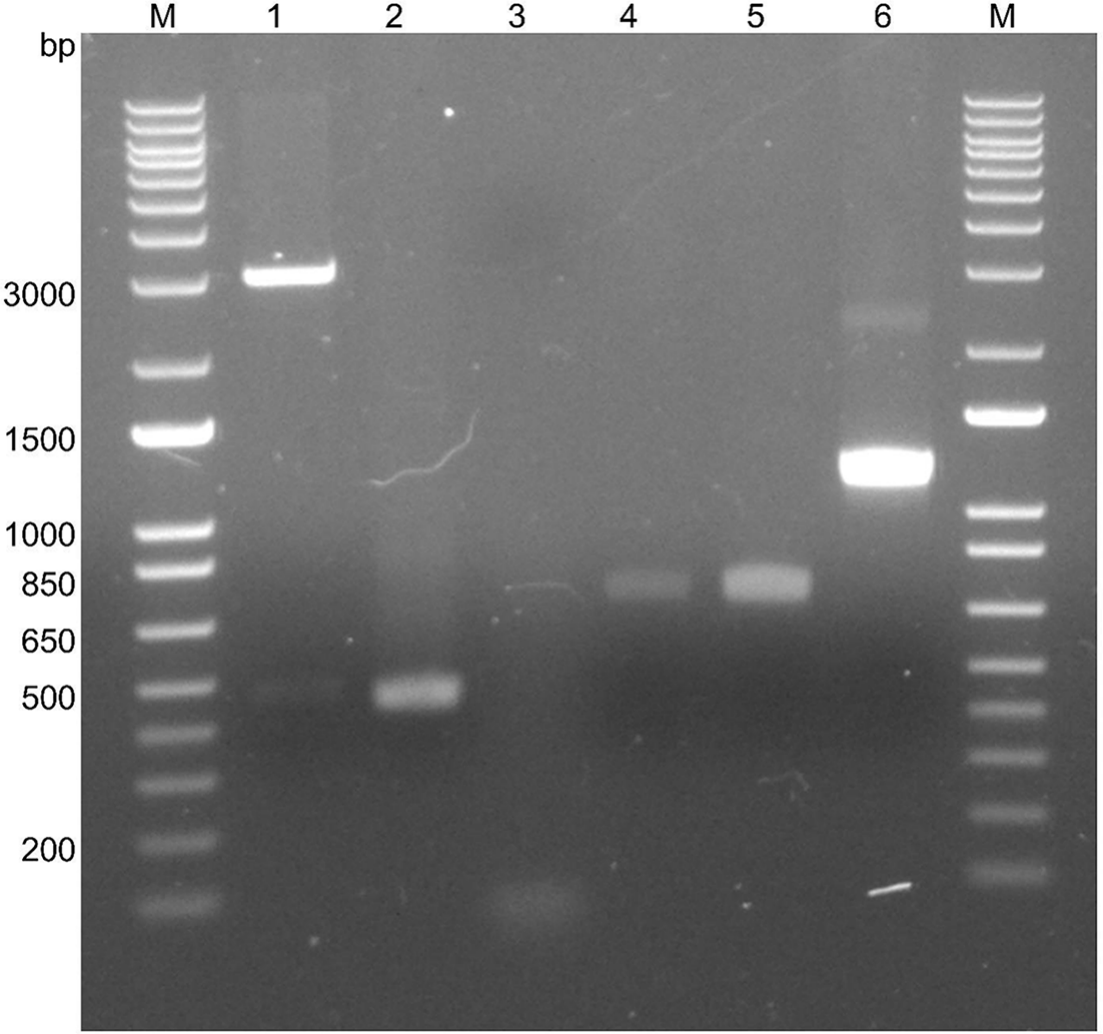
Gene cloning and expression construct. Lane 1: detection of pGEM-*pilV* of about 400 bp after digestion. Lane 2: *pilV* PCR as positive control (400 bp). Lane 3: Negative control. Lane 4: amplicon of *pilV* after PCR using M13 primers. Lane 5: amplicon of empty vector pET32b(+) (*ca*. 700 bp). Lane 6: amplicon of vector pET32b-*pilV* (*ca*. 1100 bp). M: molecular weight marker.

After cloning, the pGEM-*pilV* construct was transformed in *E. coli* JM109 cells. The recombinant plasmid was then extracted and analyzed, and the electrophoretic analysis showed fragments of 400 bp of pGEM-*pilV* (Fig. 1). The presence of the recombinant plasmid was confirmed a second time by PCR using M13 primers, amplifying the pGEM-*pilV*; we obtained a fragment of 800 bp (Fig. 1). Thus, cloning was successful and the construct was independently subcloned into the recombinant plasmid pET-32b(+).

### Subcloning *pilV* into pET-32b(+) Expression Vector

The *pilV* insert was subcloned into the expression vector pET-32b(+). Then the plasmid DNAs were extracted and purified (miniprep) and PCR were done using T7 primers. The gel analysis showed the corresponding bands of the plasmids with the pilins and of pET-32b(+) without any pilin insert (Fig. 1). Then the inserts were sequenced, and the obtained sequences were translated. Comparing the translated sequences of *pilV* from the corresponding plasmids with the reported GenBank sequence of PilV (AWP39907.1) confirmed that the mature proteins are identical (data not shown).

### Expression and Purification of PilV

**Fig. 2:**
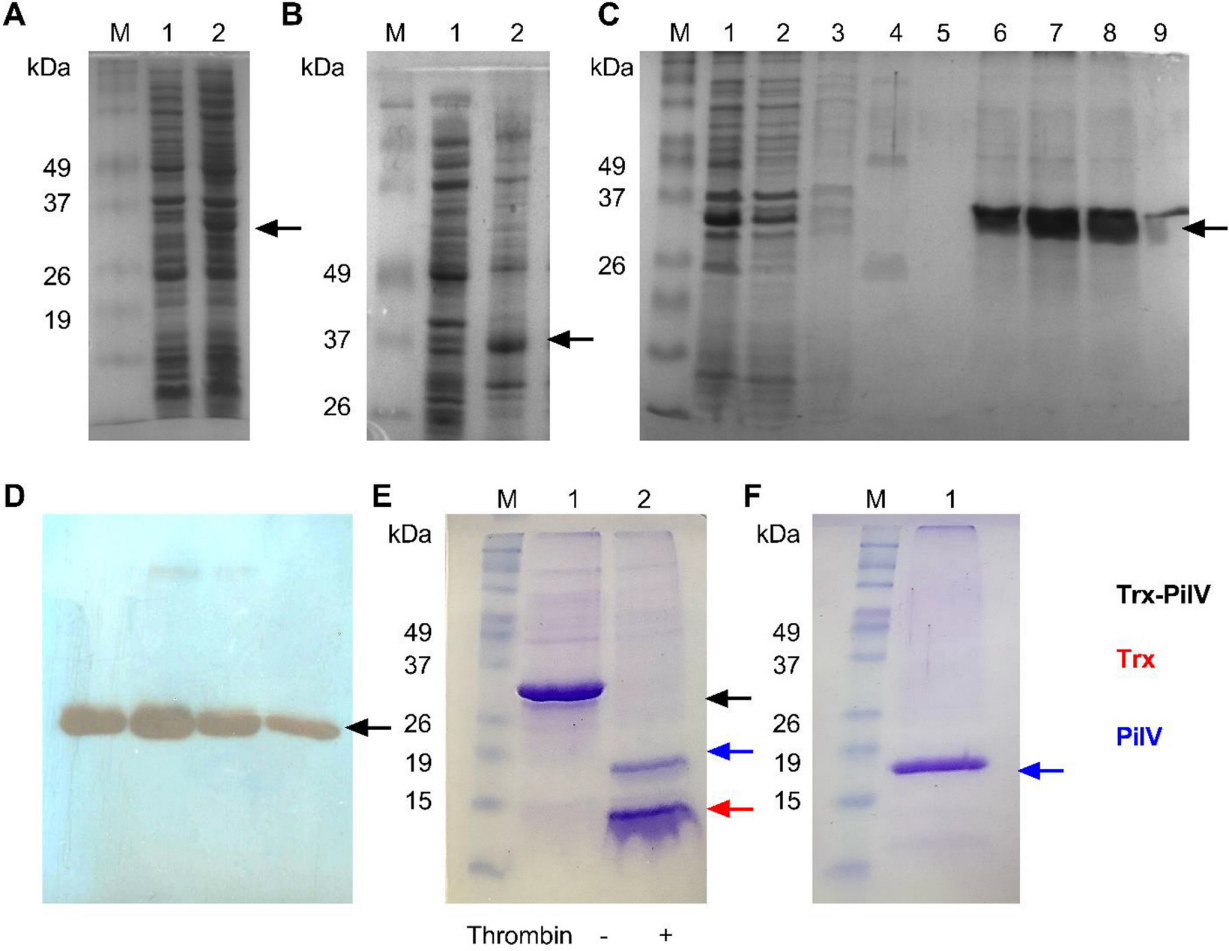
Expression and purification of PilV of *Acidithiobacillus thiooxidans*. (**A**) Heterologous expression of PilV in *E. coli* BL21-CodonPlus (DE3)-RIPL with the pET32-*pilV* vector. M: molecular weight. Lane 1: protein profile before induction. Lane 2: protein profile after induction. (**B**) Solubilization of PilV with detergent. Lane 1: soluble fraction. Lane 2: fraction solubilized with detergent. (**C**) Purification of PilV by nickel affinity chromatography. Lane 1: solubilized fraction. Lane 2: unbound fraction. Lane 3: proteins eluted with buffer C. Lanes 4 and 5: protein eluted with buffer C supplemented with 10 mM imidazole. Lanes 6 to 9: proteins eluted with buffer C supplemented with 300 mM imidazole. (**D**) WB detection of fractions eluted with 300 mM imidazole. (**E**) Cleavage of the Trx-PilV fusion protein with thrombin protease. M: molecular weight. Lane 1: fusion protein without protease (-). Lanes 2: cleaved fusion protein after incubation with thrombin protease (+). Trx: 13.9 kDa and PilV: 19.2 kDa. (**F**) PilV protein obtained after purification by anion exchange chromatography. M: molecular weight. Lane 1: purified PilV.

Initially, attempts were made to express the PilV protein as a fusion protein together with the Trx protein in *E. coli* BL21 (DE3) strain; however, there was no correct expression of this despite changing different parameters (*e.g.*, expression time, temperature, and inducer concentration; data not shown). So, we decided to analyze whether *pilV* had a “bias codon”. This analysis showed us that *pilV* has 6 low-use codons that correspond to 3 prolines (CCC), 2 isoleucines (AUA), and one arginine (CGA). Considering this, we decided to use the *E. coli* BL21-CodonPlus (DE3)-RIPL cells, obtaining a correct expression of the fusion protein with an expected theoretical weight of 33 kDa (Fig. 2A).

The solubility tests indicated that the fusion protein is in the insoluble fraction, probably due to an interaction of the α-helix of the N-terminal region of PilV with the cell membrane since this α-helix is transmembranal according to the MEMSAT-SVM analysis from PSIPRED and DeepTMHMM. Therefore, we decided to use the Triton-X100 detergent at a percentage of 2% v/v, managing to solubilize the fusion protein (Fig. 2B), to then perform a nickel affinity chromatography, since the fusion protein has a 6x-His tag before the thrombin cleavage site between the Trx protein and PilV (Fig. 2C). The results show that in fractions 6-9, the fusion protein elutes at the expected molecular weight, which is confirmed by WB (Fig. 2D). These fractions were combined and desalted to be digested with thrombin protease and to have PilV without the Trx tag (Fig. 2E). Using the thrombin assay at a concentration of at least 1 U/ml of sample, a good quantity of PilV can be obtained for its later purification. A screening was conducted to determine the NaCl concentrations at which Trx does not bind but PilV does, resulting in 150 mM NaCl. Consequently, the protein sample was desalted into buffer (25 mM Tris-HCl pH 8.0, 150 mM NaCl, 2% Triton X-100) before washing the column with 25 mM Tris-HCl, 150 mM NaCl, 2% CHAPS, and finally the protein was eluted using the same buffer supplemented with 300 mM NaCl (Fig. 2F).

### Structural Model of PilV of *A. thiooxidans*

**Fig. 3:**
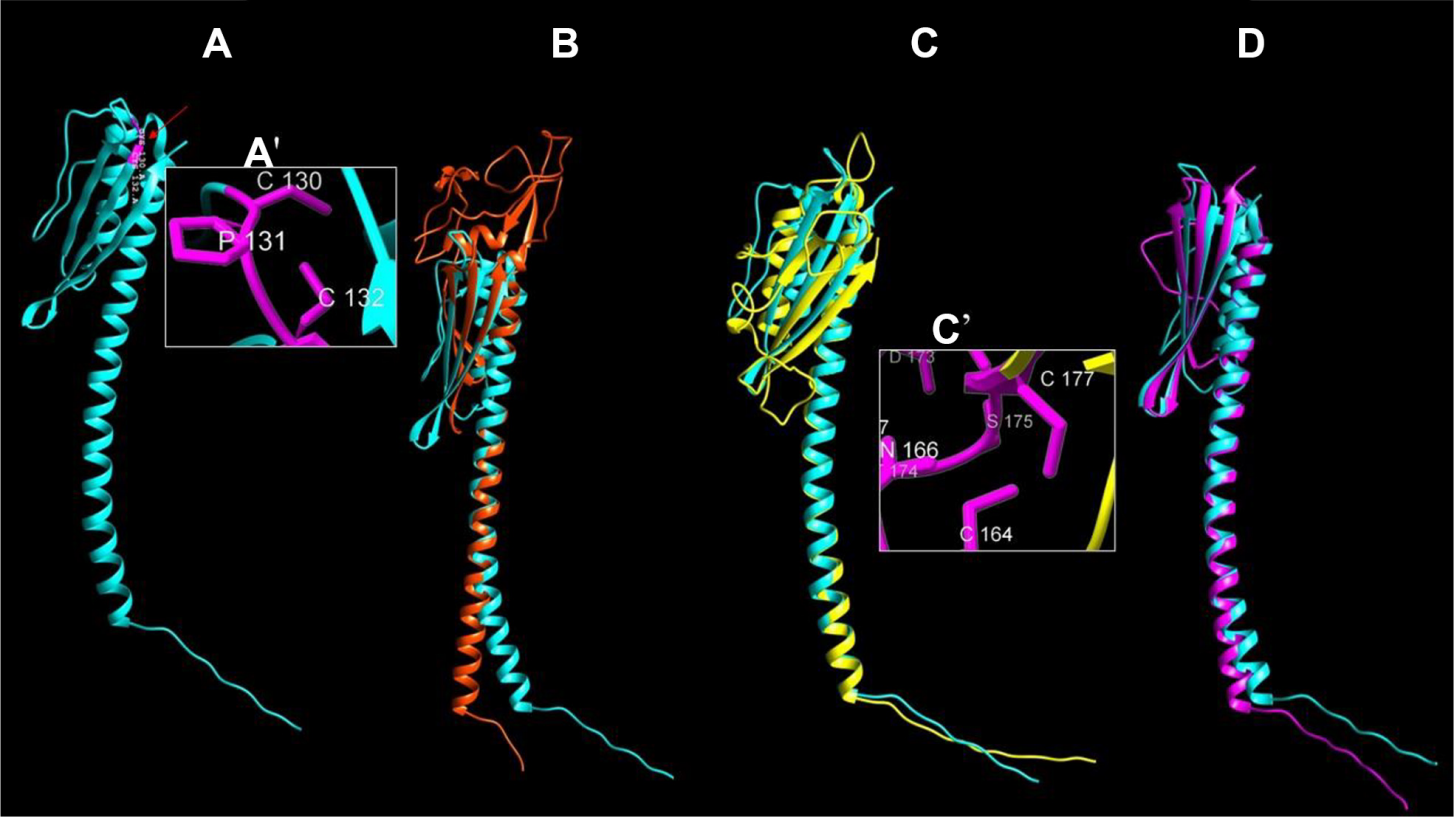
Predicted structure of: (**A**) PilV of *A. thiooxidans* (cyan color) obtained by AlphaFold2 and compared through superposition with: (**B**) “prepilin-like protein” of *T. thermophilus* HB27 (orange; GenBank: AAM55483.1); (**C**) “type 4 fimbrial biogenesis protein PilV” of *P. aeruginosa* PAO1 (yellow; NP_253241.1); (**C'**) shows its D-region, and (**D**) “type IV pilus minor pilin PilV” of *G. sulfurreducens* PCA (magenta; AAR34389.2). PilV structures were overlain (two by two) using UCSF Chimera. Red arrow in (**A**) indicates the putative D-region, amplified in (**A'**) to show the two Cys residues separated by Pro-131.

The predicted structure of PilV of *A. thiooxidans* obtained using AlphaFolfd2 shows the typical characteristics of pilin: a hydrophobic α-helix of stick-like shape in its α1-N (Leu-13 to Gly-44), a less hydrophobic α1-C (Asn-45 to Phe-71), and β-sheets joined with head-like loops and two terminal cysteines (Cys-130 and Cys-132) flanking a putative D-region; these two Cys are very close (Fig. 3A and 3A') and hinder the formation of the typical C-terminal disulfide bond (Fig. 3B and 3C'). The C-terminal head-like structure includes the characteristic short αβ-loop (between α1-C and the first β-sheet) of amino acids (Asn-72 to Ala-115) (Fig. S1).

Once the PilV model of *A. thiooxidans* was selected, we aligned the structure against the corresponding structures of *T. thermophilus* HB27, *P. aeruginosa* PAO1 and *G. sulfurreducens*. The PilV of *A. thiooxidans* overlaps PilV of *P. aeruginosa* (Fig. 3C) and of *G. sulfurreducens* better, with which it has 15.92% identity (RMSD between 61 pruned atom pairs of 1.030 Å across all 153 pairs: 9.067) and 16.67% identity (RMSD between 78 pruned atom pairs of 0.889 Å; across all 130 pairs: 5.471) respectively. Interestingly, the alignment of PilV of *G. sulfurreducens* and *A. thiooxidans* shows a major identity and superposition in both the stick-like and head-like shape structures (Fig. 3D). The PilV of *A. thiooxidans* and *T. thermophilus* showed the lowest identity and superposition, 7.64%(RMSD of 34 pruned atom pairs is 0.831 Å; across all 137 pairs: 18.501).

### PilV Behavior at Different pH

To investigate the behavior of the protein at different pH values, it is necessary to evaluate the protonation state of the titratable groups under conditions in which the electrostatic environment changes. After obtaining the different states of protonation of PilV at pH 2, 3, 4, 5, 6, 7, and 8, molecular dynamics simulations were carried out for each pH at 300 K temperature (26.8°C).

**Fig. 4:**
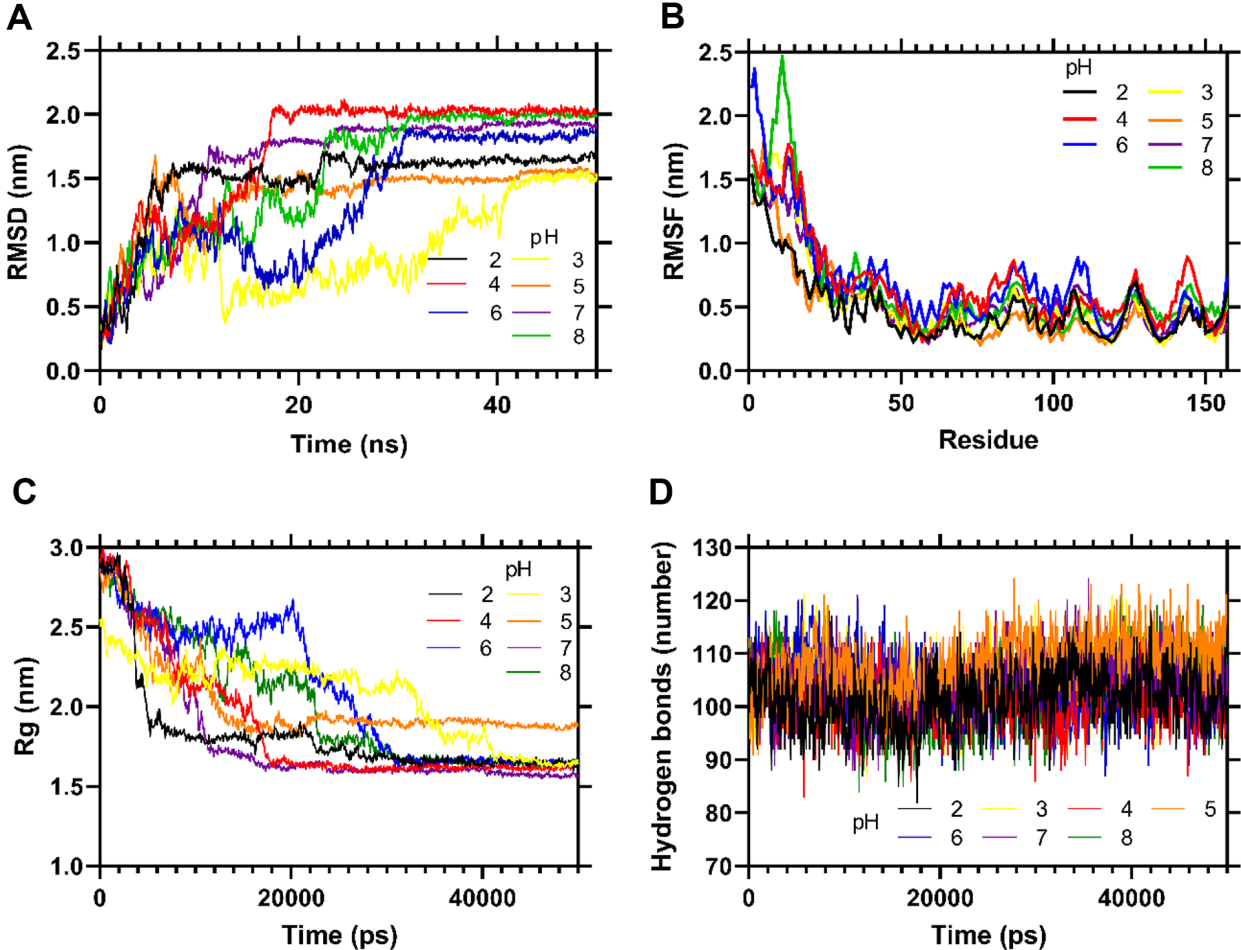
pH-MD simulations of PilV. (**A**) RMSD. (**B**) RMSF. (**C**) Rg. (**D**) Hydrogen bond plot of PilV at different pH.

The RMSD for PilV indicates a deviation of 1 to 1.5 nm at all modeled pH, after 45 ns. These changes in the initial structure may be due to the fact that the hydrophobic N-terminal of PilV is exposed to the solvent, causing self-folding to decrease the contact area and exposure to the polar medium (Fig. 4A). This is verified in the RMSF graphs: the first 40 residues outside the globular domain of PilV have higher RMSF than the rest of the protein, due to the rate of fluctuations comparable at all the pH tested (Fig. 4B). In addition, it is observed that the region of residues 65-120, predicted as disordered, has a greater fluctuation rate.

The Rg analysis shows that PilV at different pH tends to compact (lower Rg), except at pH 5.0, where a small portion of the N-terminal folds in on itself. This analysis leads us to infer that PilV remains stable at different pH and shows resilience to pH changes (Fig. 4C). The number of intra-protein H-bonds, and therefore the stability of the protein in PilV, is maintained at the different pH, except for pH 5 (Fig. 4D).

**Fig. 5:**
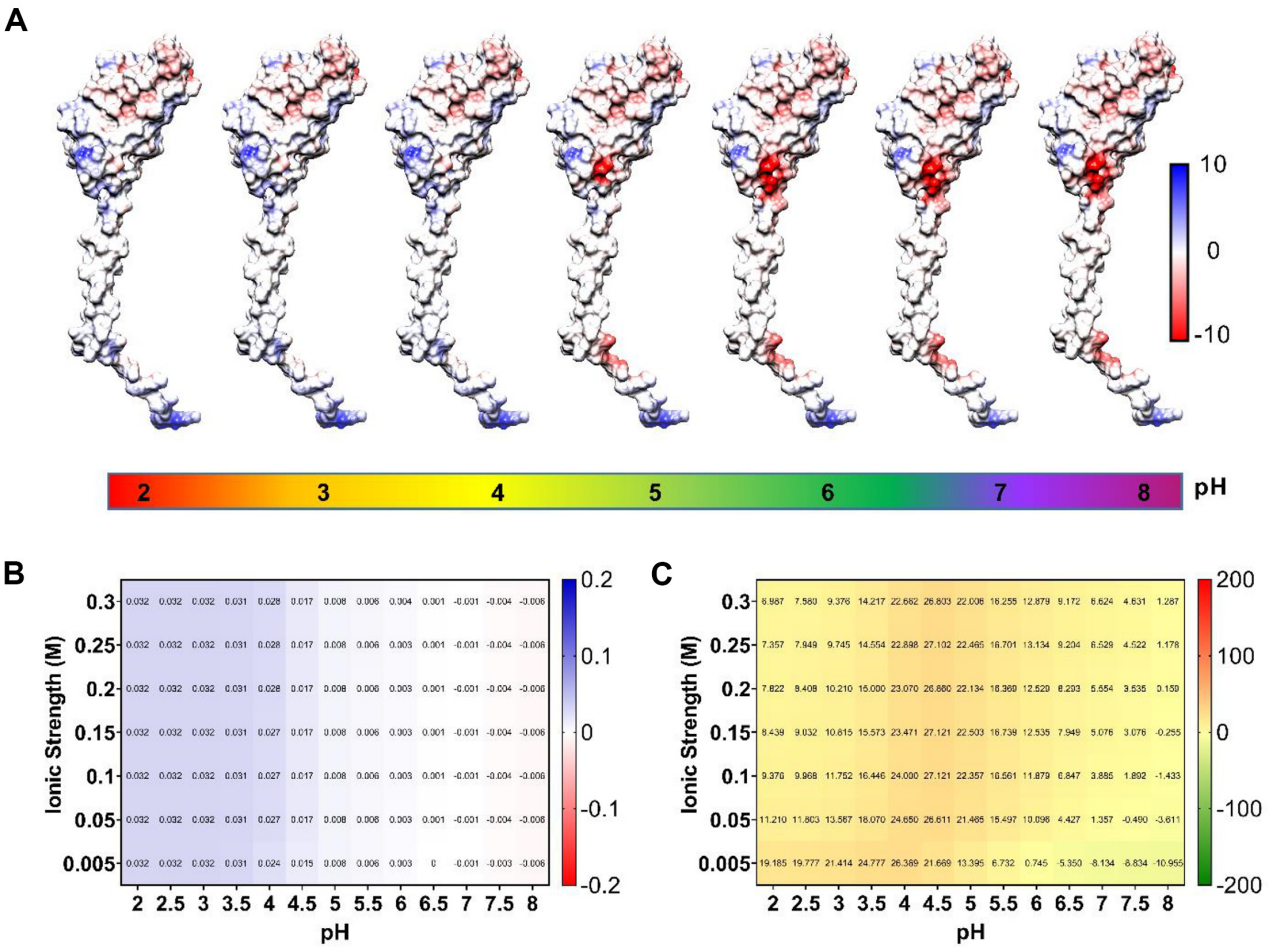
Computational characterization of PilV. (**A**) Visualization of the surface charge of PilV is achieved by incorporating amino acid protonation states calculated based on pH using PDB2PQR and APBS. (**B**) Heatmap of predicted average charge for PilV at different pH and ionic strengths. (**C**) Heatmap of predicted protein stability in the folded state, considering the interactions between ionizable head groups in J/residue for PilV at different pH and ionic strength values.

For a deeper understanding of the stability of PilV at different pH, surface electrostatics were analyzed *in silico*; the data were obtained with the APBS-PDB2PQR program. The surface charge of PilV remains homogeneous at different pH (Fig. 5A); it is observed with few regions that indicate ionizable groups, probably because this protein has 4 glutamic acid, 2 arginine and one lysine. The protein acquires little surface charge at pH <4.0 and remains neutral at pH >4.0 (Fig. 5B). Therefore, having low surface charge, it maintains structural integrity, even with different values of ionic strength (Fig. 5C). The pH that gives stability of the folded state show homogeneity.

**Fig. 6:**
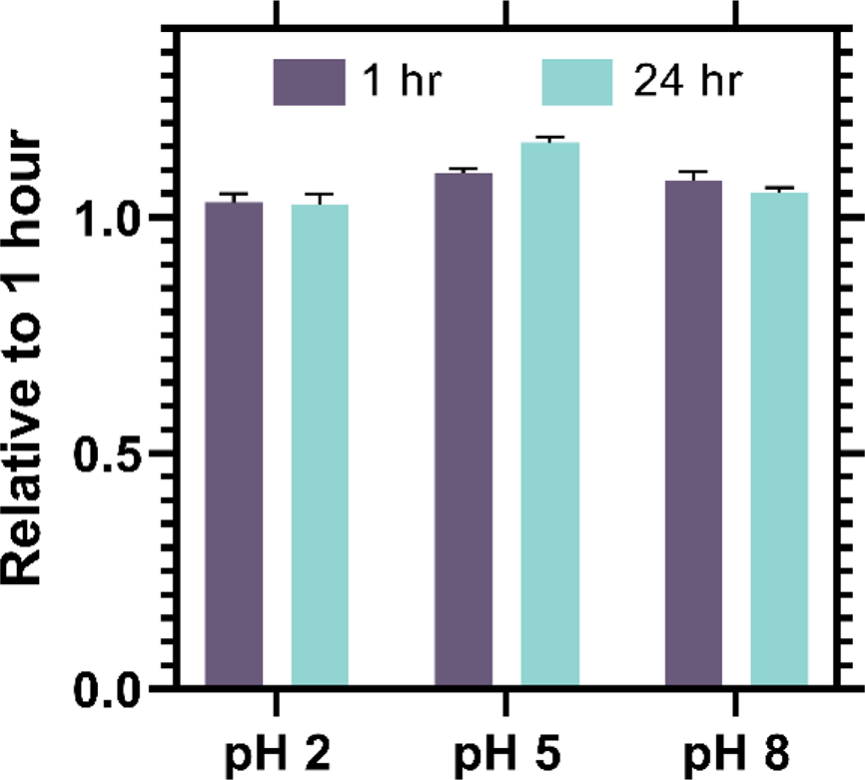
The precipitation behavior of PilV was examined by assessing its likelihood to form precipitates under three distinct pH conditions. The measurements were carried out in triplicate and then normalized to the average value obtained at the 1-hour mark. The initial protein concentration used was 1 mg/ml.

Considering that the results from molecular dynamics and folded state stability analyses demonstrate resilience to a range of pH values, we decided to experimentally assess these conditions. To do so, we evaluated the solubility of PilV at pH 2, 5, and 8 by monitoring the protein concentration over time through UV-Vis spectroscopy, measuring the absorbance at 280 nm. Following incubation of the recombinant PilV at a concentration of 1 mg/ml for a period of up to 24 h, we did not observe any precipitation, and the protein remained stable at pH 2, 5 and 8 (Fig. 6).

In conditions of extreme pH, the primary factor causing the protein to unfold is the repulsion between charged groups within the protein molecule, modifying salt bridges and hydrogen bonding formed between the side chains, leading to denaturation and subsequent aggregation and precipitation due to exposure of the hydrophobic core. For some proteins, the equilibrium between these internal repulsive forces and hydrophobic interactions (and potentially electrostatic disulfide cross-links and metal-protein interactions) is delicate enough that the protein may undergo partial unfolding or, in the most extreme scenarios, retain its folded structure [48]. So, if protein lacks charged amino acids, it may have fewer charged groups available for electrostatic interactions. As a result, the electrostatic repulsion within the protein may be reduced in extreme pH conditions. This reduction in charged groups could result in the equilibrium between repulsive and stabilizing forces shifting in favor of the latter.

### Using Intrinsic Fluorescence to Measure PilV Stability upon Different pH and Denaturing Conditions

Tryptophan fluorescence is commonly used to measure the stability of proteins by monitoring conformational changes. The fluorescence intensity and the maximum wavelength of tryptophan emission are used as signals to measure the stability of proteins. This method is particularly useful for monitoring the unfolding of proteins and can be employed to study the effect of pH and temperature on protein conformational changes [49].

**Fig. 7:**
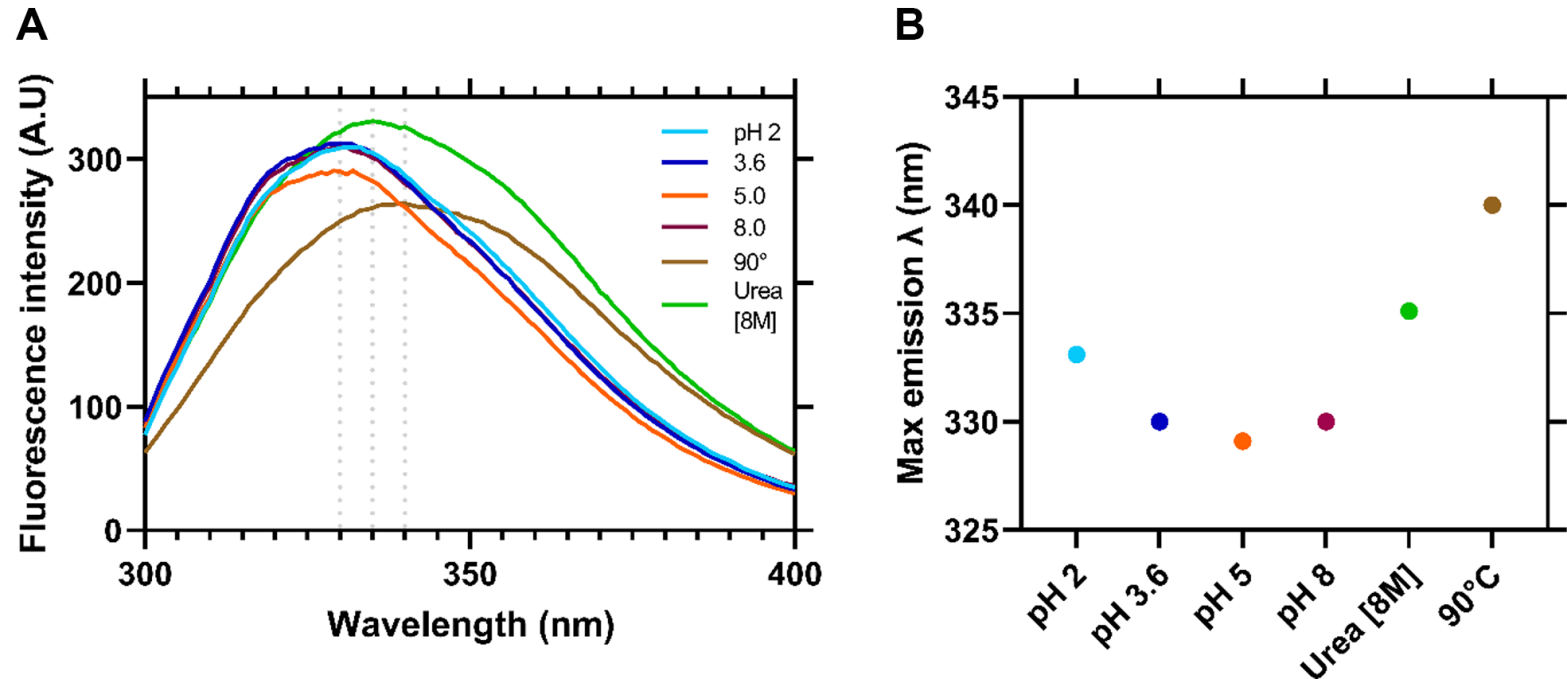
Intrinsic fluorescence spectra of PilV. (**A**) Emission spectra pf PilV at different pH and denaturing condition (chaotropic agent and high temperature, separately). An excitation wavelength of 280 nm was used and monitored emission in the range of 300-400 nm. (**B**) Max emission λ plotted with respect to pH and denaturing conditions.

Changes in the tertiary structure of PilV were monitored by analyzing changes in the intrinsic fluorescence emission. Intrinsic fluorescence spectra of PilV at different pH are shown in Fig. 7A. Regarding the maximum fluorescence intensity of PilV at different pH values, it was 330 nm for pH 3.6, 5, 8, and 333 nm for pH 2 indicating a characteristic feature of folded proteins (Fig. 7B). It's worth mentioning that the fluorescence intensity is very similar across the range of pH values, except for pH 5, which exhibits a slight decrease, but probably the microenvironment around aromatic residues was relative unchanged in different pH levels (Fig. 7A). To gain more insight into the structural stability of PilV, we denatured the protein with high concentration of urea (8 M) firstly, and then we incubated it at high temperature (90°C). For both conditions, the fluorescence spectrum shows a red shift with maximum intensities of 335 and 340 for urea and temperature, respectively (Fig. 7B), indicating that the tertiary structure of PilV destabilized due to unfolding (Fig. 7A). Remarkable, during denaturation with urea, there is an increase in fluorescence intensity, a phenomenon that has already been observed in other proteins, most likely due to the removal of other tryptophan quenching as the protein unfolds [50]. Finally, it is important to say that PilV has 2 tryptophan residues in its sequence [32].

## Discussion

Seminal reports about T4P have been based on studies only in mesophile and pathogenic bacteria such as *P. aeruginosa*, by studying the homolog expression of the pilins. Although the homologous expression has many technical complications and flats. It is easier to analyze than heterologous T4P of bacteria from extreme natural environments. Understanding of the latter remains poor because it is more difficult to find culture media and suitable conditions. Heterologous expression is therefore the best option for characterizing extremophiles.

However, it is crucial to be mindful of the inherent challenges associated with this approach, including protein stability, folding, and functional assays in non-native hosts.

In this work we present the methodology developed to achieve heterologous expression and acquire pure PilV, as well as the results obtained. Various methodologies were followed and some vector expression systems were assayed, namely the two-step cloning that enables the construct pGEM-*pilV* to be independently subcloned into the recombinant plasmid pET-32b(+).

Improving yields is an important issue in the production of recombinant proteins. One of the determining factors is the incubation temperature; we observed that low temperature yields certain advantages such as obtaining a soluble recombinant protein, avoiding the formation of inclusion bodies. This significantly improves protein quality, as was previously observed by Mühlmann *et al*. [51] and Bartolo-Aguilar *et al*. [52]. Our results show that PilV is better expressed at 16°C; despite also being expressed at 37°C, we chose to express PilV at the lower temperature to obtain a well-folded protein.

We succeeded in standardizing the appropriate purification technique for PilV protein, obtaining a yield of 2.0 mg of solubilized protein in a liter of culture after 2 purification steps. Pilin PilV was expressed mainly in the insoluble fraction and recovery after detergent solubilization, likely because an interaction between the hydrophobic α-helix of the N-terminal of PilV and the bacterial membrane is broken: PilV can be considered a transiently peripheral protein, as pilus assembly involves the transport of pilins through the membrane; first, pilin precursors present in the cytoplasm are transported to the outer membrane through the SecA translocon [53], then peripheral membrane protein can associate with the membrane in a variety of ways. For example, the mitochondrial enzyme dihydroorotate dehydrogenase (DHODH) has an N-terminal transmembrane helix to ensure correct orientation of the substrate binding site towards the membrane; of these, DHODH requires detergent micelles to stabilize it before further experimental characterization such as native mass spectrometry [54].

The expression and purification of PilV of *A. thiooxidans* enables techniques such as protein crystallization to be developed, to expand knowledge about this pilin; e.g., to elucidate its structure and function. However, currently the use of simulations is transforming molecular research through modeling systems for the initial approach. Thanks to this, it is possible to advance in molecular characterization and subsequently recreate experiments to validate the predicted characteristics. Consequently, and since our cloning was successful (Fig. 1), we decided to model the PilV sequence using a structure approach to seek homology between the PilV of *A. thiooxidans* and three other distant taxa, taking advantage of the powerful tool AlphaFold2 to make predictions of protein structure [41] since pilins share a similar basic modular structure (Fig. 3A and 3D). Our predicted model of PilV of *A. thiooxidans* resulted in such a “lollipop-like” structure, confirming that the predicted structure is like other pilins (Fig. 3).

We aligned the structure of *A. thiooxidans* PilV with the corresponding PilV of two classes of Proteobacteria, the Gammaproteobacteria *P. aeruginosa* and the Deltaproteobacteria *G. sulfurreducens*; we also compared it with PilV of the extremophile *T. thermophilus*, phylum Deinococcus-Thermus. The selected species have the PilV domains known as extracellular T4P pilus assembly proteins involved in cell motility [55] as minor pilins [56]. Despite the existence of variable regions, the PilV model of *A. thiooxidans* and the selected species confirm the canonical basic architecture of T4P pilins. The primary structure of the compared PilV proteins have low similarity, <16.67%; nevertheless, the results of superimposed PilV structures or tertiary structure (Fig. 3) exhibit a greater overlap along the N-terminal α-helix, with the lowest identity because of the variable regions beyond the first 25 amino acids, commonly observed in evolutionarily distant species [5].

It is known that the greatest structural difference in the minor pilins is the versatile αβ-loop region that connects the α1-C helix to the β-sheet, as well as the D-region or the disulfide-bonded loop enclosed by the C-terminal disulfide bond that is found in some species [7] (Fig. 3C'). Disorder analysis shows that the entire αβ-loop region is located precisely at the peak of the most disordered region of PilV of *A. thiooxidans*, but it could transition into an ordered state [32]. Additionally, it has been observed that the αβ-loop is involved in the proper assembly of pilins and exhibits a slow structural dynamic (μs to ms) [57], suggesting its role in protein-protein interactions. In both major and minor pilins, the D-region typically consists of 10 to 20 amino acids forming a loop (Fig. 3). It is proposed that this contributes to the structural stability of the globular domain, and it is often found connected to the last β-sheet of the tail [58], stabilizing the interactions between structural pilins [59] or exposed for surface binding and pilus assembly [60]. It should be noted that these disulfide bridges are disrupted under reducing conditions, leading to the rapid disintegration of the pilus structure [61].

Surprisingly, although PilV of *A. thiooxidans* possesses two Cys in its C-terminal, they do not form a disulfide bond with each other due to their close position (130 and 132) and the presence of a Pro residue between them (Pro131; Fig. 3A'). When two consecutive Cys residues are separated by a Pro residue, it can lead to the formation of a small disulfide loop, but the formation of a disulfide in Cys-Cys requires prior formation of an unfavorable cis peptide bond between the cysteines, and prolines have only limited ability to adopt the *cis* configuration [62]. Like PilV of *A. thiooxidans*, the PilV of *G. sulfurreducens* do not show a D-region, while the PilV of *P. aeruginosa* and *T. thermophilus* do have D-region (Cys164 to Cys177 and Cys170 to Cys185, respectively), forming a disulfide bond. These results confirm that the D-region in minor pilins is variable and not always present [6]. Similar to FimA of *D. nodosus*, in our PilV model we found two hydrogen bonds joined to the last β-sheet with Cys 130 (data not shown) [63]. This could imply that hydrogen bonds are responsible for the intermolecular interactions.

Considering this information, we can assume that disulfide bonds are not formed in D-region between the two cysteines of PilV of *A. thiooxidans* but rather with the cysteines of another minor pilin (PilW, PilX or PilY1) or that they serve as a surface sensor or complex because D-region is exposed to the surface; *e.g.*, a divalent metal of metal sulfide such as pyrite (FeS_2_) forming a Cys-S-Fe complex as suggested by Rojas-Chapana and Tributsch [64]. Moreover, according to Ye *et al*. [65] the electron transferred is through the disulfide bridges, promoting the transfer of S species to the sulfur-oxidant bacteria.

Previously we reported that the pili of *A. thiooxidans* exhibit characteristics that would make them resilient to changes in pH and reducing environments [32]. Here, specifically for the PilV pilin, we evaluated specific regions of the tertiary structure that are known to be in contact with the external environment. In the case of the segment corresponding to the αβ-loop (from Asn-72 to Ala-115), we found that compared to the same region of PilV in *P. aeruginosa*, the degree of intrinsic disorder has opposite values. The αβ-loop of *A. thiooxidans* shows a high tendency towards disorder, whereas that of *P. aeruginosa* shows a strong tendency towards order. For the case of the same region of PilV in *T. thermophilus*, something very similar occurs in the central structure of the loop; however, at the ends, a pronounced tendency toward disorder is observed. In the case of PilV of *G. sulfurreducens*, due to the size of their αβ-loop (24 amino acids) it is not possible to determine a disorder region, however the middle αβ-loop region shows a tendency to disorder (Fig. S2). For the αβ-loop segments in particular, these results confirm our previously reported data [32].

We have proposed a model by which the extracellular pilins of *A. thiooxidans* can maintain their stability and functionality at different pH to which pilins are exposed (from their synthesis in the cytoplasm to their incorporation into the pili and coming into contact with the extracellular medium). Until the present study, the effect of pH on the minor pilins had not been investigated nor, to the best of our knowledge, has any previous computational study investigated the behavior of PilV at different pH. Here, the computational characterization was performed using molecular dynamics, and the analysis revealed the stability of PilV at different pH. Other proteins become unstable to changes of pH due to ionizable side chains and Coulomb repulsion between the polar groups of the charged heads of amino acids (*e.g.*, Asp, Glu, His, Lys, and Arg) [66-68]. Analysis of the amino acid composition of PilV of *A. thiooxidans* indicates a significant decrease in charged amino acids, which most likely confers acid stability. Indeed, the electrostatic repulsion between residues with the same charge is one of the main factors affecting protein structure, stability, and function [69-71].

If we observe the surface charge at different pH values, we notice that overall PilV maintains a low net charge and very similar values for the stability of the folded state. We did not carry out further analyses under more basic pH. These predictions were initially confirmed by measuring the protein’s tendency to precipitate, which indicated that it remains soluble even with significant changes of pH (Fig. 6). Further biophysical characterization using intrinsic tryptophan fluorescence provides information about the local microenvironment of the tryptophan residue, which responds very sensitively to any change in the protein’s tertiary structure. The maximum fluorescent intensity of PilV at different pH values is around 330 nm, suggesting that this residue stays in a hydrophobic environment. There is only a red shift when the protein is exposed to a high concentration of urea or when it is incubated at a high temperature, indicating that the tryptophan residues’ microenvironment changes from a non-polar to a solvent-exposed environment during the unfolding of the protein.

In summary, despite the existence of various methods of protein cloning and purification, few reports have been published on acidophilic chemolithoautotrophs, which are of interest due to their extracellular protein present in protonated microenvironments that do not resemble heterologous organism such as *E. coli*. We proposed such method for cloning and heterologous expression. To date, there have been very few reports in the literature reporting the study of pilins from non-pathogenic bacteria. It is crucial to begin characterizing pilins from different organisms, as they can provide us with guidance for applying their key adaptability characteristics in various conditions. As demonstrated by our study of pilin PilV, we suggest that due to its unique amino acid composition, it exhibits acid stability, which could have significant implications in medicine and in biotechnology such as bioleaching of sulfide mineral. For instance, identifying stabilizing substitutions; some proteins may find stabilizing amino acid substitutions by comparing their sequences with homologs from acidophilic microorganisms. A bioinformatics approach makes it possible to explore experimental conditions that close the gap and guide the characterization toward realistic achievements.
